# Plasma nesfatin-1 level is associated with severity of depression in Chinese depressive patients

**DOI:** 10.1186/s12888-018-1672-4

**Published:** 2018-04-03

**Authors:** Min-Min Xiao, Jiang-Bo Li, Lan-Lan Jiang, Hui Shao, Bao-Long Wang

**Affiliations:** 1 Clinical Laboratory, Affiliated Provincial Hospital of Anhui Medical University, 17 Lujiang Road, Hefei, 230001 Anhui China; 2 Clinical Laboratory, The Second People’s Hospital of Wuhu City, Wuhu, 241001 Anhui China; 3 Department of Clinical Psychiatry, The Second People’s Hospital of Wuhu City, Wuhu, 241001 Anhui China

**Keywords:** Plasma nesfatin-1, Depression, Severity

## Abstract

**Background:**

Nesfatin-1 plays a role in the regulation of emotional states like depression. The aim of this study was to investigate the plasma nesfatin-1levels in Chinese patients with depression and healthy subjects, and to determine the possible association between the plasma nesfatin-1 level and the severity of depression.

**Methods:**

A total of 103 depressive patients and 32 healthy subjects were assessed. According to HAMD-17scores, 51, 18, and 34 patients were enrolled in the mild depression, moderate depression, and severe depression groups, respectively. Plasma nesfatin-1 levels were determined by the ELISA method. Differences between groups were compared and associations between plasma nesfatin-1 and other variables were analyzed.

**Results:**

The plasma nesfatin-1 was significantly positively correlated with HAMD-17 score (*r* = 0.651). Compared with healthy controls (8.11 ± 3.31 ng/mL), the plasma nesfatin-1 level significantly increased in patients with mild depression (11.17 ± 3.58 ng/mL), with moderate depression (16.33 ± 8.78 ng/mL), and with severe depression (27.65 ± 8.26 ng/mL) respectively. Plasma nesfatin-1 level (Odds ratio [OR] = 1.269) was an independent indicator for severe depression by multivariate logistic regression analysis.

**Conclusion:**

The plasma nesfatin-1 level is positively correlated with the severity of depression. Plasma nesfatin-1 level may be a potential indicator for depression severity.

## Background

Depression is a state of low mood and demotivated condition that affects a person’s feelings, cognition, and behaviors. Major depressive disorder is the most common of serious psychiatric disorders and is recognized to be a high risk factor of suicide [1]. Thus, evaluating the severity of depression is crucial for treatment [2]. In patients diagnosed with depression, Hamilton Rating Scale for depression (HAMD) is a widely used, standardized, clinician administered questionnaire to assess and rate the severity of depression. However, there are no commonly accepted depression biomarkers to improve diagnostic accuracy or to evaluate severity ratings [3]. Although the cellular and molecular mechanisms underlying the pathophysiology of depression are not been fully elucidated, there have been increasing interests in associations between depression and changes in various biochemical pathways, including inflammatory, neurotrophic and hypothalamic-pituitary-adrenal (HPA) axis alterations [4, 5].

Nesfatin-1, a newly discovered hormone, was derived from nucleobindin-2 (NUCB2). Some previous studies have reported that nesfatin-1 played a role in integrating feeding, glucose homeostasis, and energy expenditure [6, 7]. Furthermore, the nesfatin-1 role in the regulation of emotional states including anxiety and stress was also be found [8]. The plasma level and mRNA expression of nesfatin-1 were increased by acute stress in rats [9]. In fact, nesfatin-1 can activate the HPA axis, the hyperactivity of which is proposed to be among the causal factors for triggering depressive episodes [10]. Because of the dysfunction of the HPA axes, with the important role of nesfatin-1 in the pathophysiology of depression [11], we hypothesized that the plasma nesfatin-1 level may be associated with depression severity. Thus, the aim of this study was to investigate the levels of plasma nesfatin-1 in Chinese patients with depression and healthy subjects, and to determine the possible association between the plasma nesfatin-1 level and the severity of depression.

## Methods

### Study population

All subjects in the depressive group were recruited from the outpatient department of psychological consultation and treatment center, the Second People’s Hospital of Wuhu City from January 2016 to April 2017. The inclusion criteria were the following: a) HAMD-17scores > 7; b) meeting diagnostic criteria for depression according to the Diagnostic and Statistical Manual for Psychiatric Disorders-Fourth Version. The exclusion criteria were the following: a) unstable psychiatric features; b) currently suffering from a severe medical condition; c) currently pregnant or lactating; d) presence of comorbid psychotic disorder, psychotic symptoms, psychoactive substance dependency or abuse, personality disorder, or mental retardation; e) received psychotropic medication within 2 weeks; f) had a history of bariatric surgery or any gastric disease; g) diagnosed of diabetes mellitus; h) FT3, FT4 or TSH was abnormal.

All subjects in the control group were recruited from the annual health examination of the Second People’s Hospital of Wuhu City. The inclusion criteria were the following: a) no mental disorders after being evaluated by psychiatrists; b) no family history of mental disorders and no history of taking psychiatric drugs; c) HAMD-17 scores ≤7. Exclusion criteria were the following: a) history of stroke or central nervous system disease; b) pregnant or lactating women.

The protocol was approved by the Ethical Committee of the Second People’s Hospital of Wuhu City. All subjects signed written informed consent in accordance with the Declaration of Helsinki.

### Measurements

The body mass index (BMI) of all participants were calculated. All blood samples from a forearm vein were collected in the morning following one night of fasting by 5-mL tubes containing EDTA. All plasma samples were obtained after centrifugation (3000×g for 5 min at 4 °C) and stored at − 80 °C until the time of assay.

The level of plasma nesfatin-1 was measured by ELISA kit from USCN Life Science Instruments (Wuhan, China) according to the manufacturer’s instructions. Thyroid Stimulating Hormone (TSH), free triiodothyronine (FT3), and free thyroxine (FT4) were assayed using an automatic electrochemistry luminescence immunoassay system (Roche Cobas E601, Mannheim, Germany). Fasting plasma glucose (FBG), triglycerides (TG), total cholesterol (TC), low-density lipoprotein cholesterol (LDL-C), and high-density lipoprotein cholesterol (HDL-C) were measured by an automatic analyzer (Beckman AU5800, Tokyo, Japan) according to the standard techniques.

17-item Hamilton Depression Rating Scale(HAMD-17) was examined for all subjects [12]. The severity of depressive symptom was classified by the following severity range for HAMD-17 score: mild depression (8–17), moderate depression (18–24), and severe depression (> 24) [13].

### Statistical analysis

Distributions of data were tested for normality using the Shapiro-Wilk test. The continuous variables were presented as mean ± standard deviation and were analyzed with the one-way ANOVA. The Chi-squared test was employed for percentages of variables. Relationships between plasma nesfatin-1 and other variables were analyzed by Spearman correlation analysis and the independent relationships were determined by multivariate linear regression analysis. A multivariate logistic regression analysis was performed to validate the risk factors of depression severity. The receiver operating characteristic (ROC) curve analysis was used to determine the cut-off value of plasma nesfatin-1. Statistical analysis was performed using the SPSS 13.0 software package, and R version 3.3.2 (http:///www.r-project.org/). Furthermore, *P*-values (two-sided) < 0.05 were considered to be statistically significant.

## Results

The study population was composed of 103 depressive patients (44 male, 59 female) and 32 healthy individuals (12 male, 20 female). Demographic variables and biochemical values of depressive group and control group are shown in Table 1. Differences between the groups were not statistically significant in terms of age, gender and BMI. Compared with the healthy controls, depressive patients had higher FBG, higher TG, higher TSH, and lower HDL-C (all *P* < 0.05). The mean plasma nesfatin-1 level in the depressive patients was 17.52 ± 9.79 ng/mL, whereas it was 8.11 ± 3.31 ng/mL in the healthy controls. Difference of mean plasma nesfatin-1 level between groups was statistically significant (*P* < 0.001, Table 1). The mean HAMD-17 scores were statistically higher in patients with depression than that in the control group (19.2 ± 8.4 in depressive group vs. 4.2 ± 0.9 in control group, *P* < 0.001).

**Table 1:** Comparison of mean values (or ratios) of study variables in depressive group and control group

Variables	Depressive group (*n* = 103)	Control group (*n* = 32)	*P* value
Age (years)	53.3 ± 9.9	51.8 ± 9.5	0.447
Gender (female/male)	59/44	20/12	0.601^a^
Duration of depression (years)	7.4 ± 4.9	–	
BMI (kg/m^2^)	22.49 ± 3.25	22.32 ± 2.45	0.782
HAMD-17 score	19.2 ± 8.4	4.2 ± 0.9	**< 0.001**
FBG (mmol/L)	6.10 ± 1.25	4.86 ± 0.49	**< 0.001**
TG (mmol/L)	1.78 ± 1.22	1.21 ± 0.54	**0.012**
TC (mmol/L)	4.61 ± 1.14	4.57 ± 0.92	0.845
HDL-C (mmol/L)	1.23 ± 0.51	1.64 ± 0.47	**< 0.001**
LDL-C (mmol/L)	2.51 ± 0.83	2.39 ± 0.79	0.509
TSH (mIU/L)	2.81 ± 0.79	2.40 ± 0.61	**0.009**
FT3 (pmol/L)	4.55 ± 0.56	4.49 ± 0.58	0.615
FT4 (pmol/L)	12.22 ± 1.54	12.56 ± 1.68	0.847
Nesfatin-1 (ng/mL)	17.52 ± 9.79	8.11 ± 3.31	**< 0.001**

^a^Calculated by Chi-squared test

**Fig. 1:**
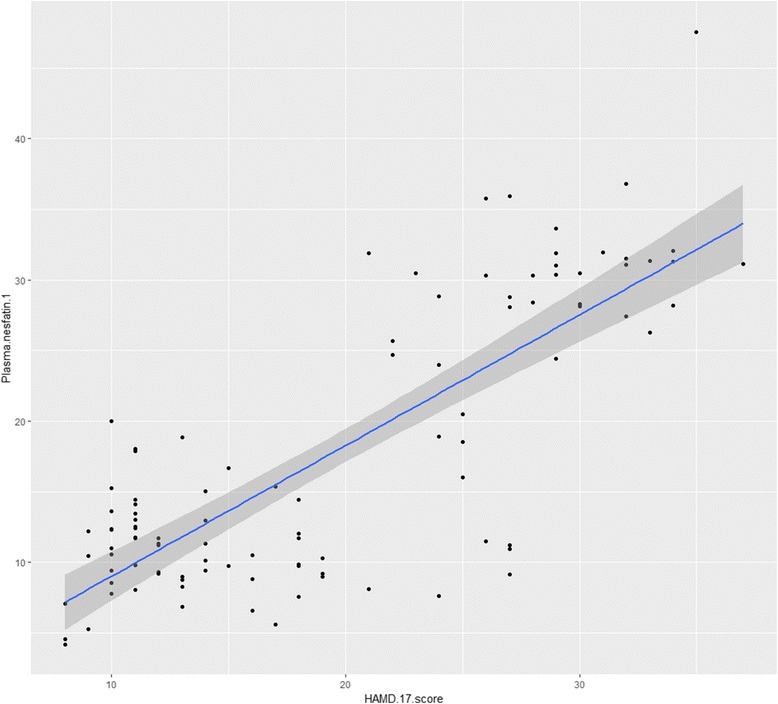
Correlation between plasma nesfatin-1 levels and HAMD-17 scores (Linear Regression)

By Spearman correlation analysis, the plasma nesfatin-1 level was significantly correlated with age (*r* = − 0.287, *P* = 0.003), duration of depression (*r* = 0.302, *P* = 0.002), BMI (*r* = − 0.305, *P* = 0.002), FBG (*r* = − 0.287, *P* = 0.003), TC (*r* = 0.254, *P* = 0.010), HDL-C (*r* = 0.232, *P* = 0.019), TSH (*r* = 0.350, *P* < 0.001), and HAMD-17 score (*r* = 0.651, *P* < 0.001, Fig. 1) in the depressive patients. Multivariate linear regression analysis showed that plasma nesfatin-1 level was negatively associated with age, whereas that was positively associated with duration of depression, HAMD-17score, and TSH (Table 2).

**Table 2:** Multivariate linear regression analysis between plasma nesfatin-1 (dependent variable) and clinical characteristics (independent variables)

	β (95%confidence interval)	SE	*P* value
Age	−0.120 (− 0.238 to − 0.002)	0.059	**0.047**
Gender	0.459 (−1.981 to 2.900)	1.228	0.709
Duration of depression	0.507 (0.301 to 0.752)	0.114	**< 0.001**
BMI	0.174 (−0.226 to 0.574)	0.201	0.389
HAMD-17 score	0.715 (0.550 to 0880)	0.083	**< 0.001**
FBG	−0.886 (−1.842 to 0.070)	0.481	0.069
TG	0.583 (−0.309 to1.476)	0.449	0.198
TC	0.965 (−0.262 to 2.192)	0.617	0.122
HDL-C	1.957 (−0.454 to 4.368)	1.213	0.110
LDL-C	−1.336 (−3.097 to 0.425)	0.886	0.135
TSH	2.173 (0.718 to3.629)	0.732	**0.004**
FT3	−0.718 (−2.677 to 1.242)	0.986	0.469
FT4	−0.360 (−1.095 to 0.374)	0.370	0.332

**Fig. 2:**
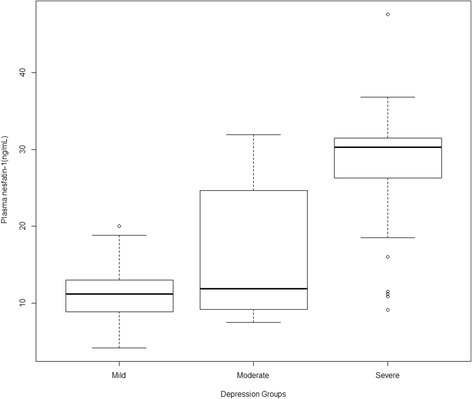
Plasma nesfatin-1 (mean ± SD) in the mild depression group, the moderate depression group, and the severe depression group

The level of plasma nesfatin-1 in females (*n* = 59) was significantly higher than that in males (*n* = 44) (19.37 ± 10.05 vs. 15.04 ± 8.96, *P =* 0.023). Among 103 depressive patients, 51 patients (49.5%) had mild depression, 18 patients (17.5%) had moderate depression, and 34 patients (33.0%) had severe depression respectively. Figure 2 shows that a significant increased trend of the plasma nesfatin-1 level among mild depressive patients (11.17 ± 3.58 ng/mL) compared to moderate depressive patients (16.33 ± 8.78 ng/mL) (*P* = 0.005), and moderate depressive patients compared to severe depressive patients (27.65 ± 8.26 ng/mL) (*P* < 0.001).

**Fig. 3:**
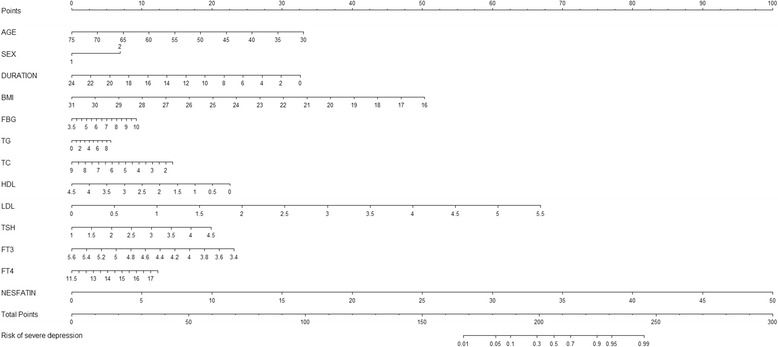
Risk factors of severe depression nomogram. (Code of sex [1: male, 2: female]) (To use the nomogram, an individual patient’s value is located on each variable axis, and a line is drawn upward to determine the number of points received for each variable value. The sum of these numbers is located on the Total Points axis, and a line is drawn downward to the Risk of severe depression axes to determine the severe depression risk)

**Fig. 4:**
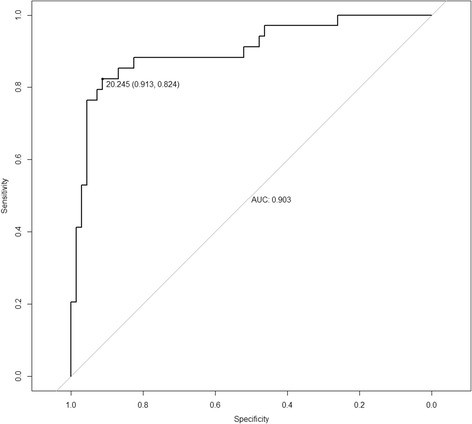
Receiver operating characteristic (ROC) curve of plasma nesfatin-1 in identification of the patients with severe depression

By multivariate logistic regression analysis, BMI (OR = 0.671, *P* = 0.017), plasma nesfatin-1 (OR = 1.269, *P* = 0.001) were the independent indicators for severe depression in the depressive patients (Fig. 3). Based on the ROC curve analysis, plasma nesfatin-1 cut-off point of 20.25 ng/mL showed 82.4% sensitivity and 91.3% specificity, and with the Area Under Curve (AUC) 0.903 (95% CI 0.835–0.971) was the optimal cut-off point for identification of severe depression (Fig. 4).

## Discussion

Recently, nesfatin-1 has been implicated in the regulation of anxiety and depression in humans [14, 15]. In this study, the plasma nesfatin-1 levels were measured in depressive patients and healthy controls in China. According to our results, the plasma nesfatin-1 level was significantly increased in depressive patients, most notably in patients with severe depression, compared to healthy controls. Our finding is in agreement with the previous report [16].

There are some studies suggesting that serum nesfatin-1 levels were significantly lower in patients with type 2 diabetes mellitus compared to healthy subjects [17, 18]. Considering the effect of high blood sugar on nesfatin-1 level, we excluded patients with diabetes mellitus in our study. A positive correlation between plasma nesfatin-1and HDL-C in the depressive patients was observed in present study, and this relationship had been previously reported by Li et al. [19] in the diabetic patients. However, the mechanism and physiological significance of this relationship is unclear. Some studies indicate that thyroid dysfunction is associate with the mood disorders and particularly with depression [20, 21]. The symptomatic of depression could be improved through the levothyroxine replacement therapy [22]. In addition, the relationship between nesfatin-1 and thyroid function had been reported in previous literature [23]. Thus, the patients with abnormal TSH, FT3 and FT4 were excluded in our study. We also observed a statistically significant association between TSH and plasma nesfatin-1 by multivariate linear regression analysis.

In the present study, plasma nesfatin-1 was significantly correlated with HAMD-17 score, furthermore, we observed the level of plasma nesfatin-1 increased gradually from mild depression to moderate depression, and from moderate depression to severe depression. This increased trend suggests that plasma nesfatin-1 is associated with severity of depression. Multivariate logistic regression analysis identified that plasma nesfatin-1 was an indicator for severe depression in our study population. In addition, the patients with severe depression could be identified with a sensitivity of 82.4% at specificity of 91.3% by plasma nesfatin-1 (cut-off value = 20.25 ng/mL). Therefore, the plasma nesfatin-1 level may be considered as a biomarker to identify Chinese patients who have severe depression.

Several limitations in this study should also be acknowledged. First, all subjects were collected from a single hospital. Second, the future studies are needed to identify the role of plasma nesfatin-1 in the progression of depression. Additionally, although all drugs had been discontinued at least 2 weeks before measurement of plasma nesfatin-1, the long-term effects of previous drugs on plasma nesfatin-1 remains unknown.

## Conclusions

The level of plasma nesfatin-1 was positively correlated with depression severity. Plasma nesfatin-1 level may be a potential indicator for depression severity. Multicentric and longitudinal studies are clearly required to validate an association between plasma nesfatin-1 level and depression severity.

## References

[1] Mogi T, Toda H, Yoshino A (2017). Clinical characteristics of patients with diagnostic uncertainty of major depressive disorder. Asian J Psychiatr.

[2] Setoyama D, Kato TA, Hashimoto R, Kunugi H, Hattori K, Hayakawa K (2016). Plasma metabolites predict severity of depression and suicidal ideation in psychiatric patients-a multicenter pilot analysis. PLoS One.

[3] Huang TL, Lin CC (2015). Advances in biomarkers of major depressive disorder. Adv Clin Chem.

[4] Cenik B, Cenik C, Snyder MP, Brown ES (2017). Plasma sterols and depressive symptom severity in a population-based cohort. PLoS One.

[5] Lamers F, Milaneschi Y, de Jonge P, Giltay EJ, Penninx BWJH. Metabolic and inflammatory markers: associations with individual depressive symptoms. Psychol Med. 2017; doi: 10.1017/S0033291717002483. 10.1017/S0033291717002483 28889804

[6] Aydin S (2013). Role of NUCB2/nesfatin-1 as a possible biomarker. Curr Pharm Des.

[7] Çelik F, Belviranli M, Okudan N (2016). Circulating levels of leptin, nesfatin-1 and kisspeptin in postmenopausal obese women. Arch Physiol Biochem.

[8] Emmerzaal TL, Kozicz T (2013). Nesfatin-1; implication in stress and stress-associated anxiety and depression. Curr Pharm Des.

[9] Xu YY, Ge JF, Qin G, Peng YN, Zhang CF, Liu XR (2015). Acute, but not chronic, stress increased the plasma concentration and hypothalamic mRNA expression of NUCB2/nesfatin-1 in rats. Neuropeptides.

[10] Könczöl K, Bodnár I, Zelena D, Pintér O, Papp RS, Palkovits M (2010). Nesfatin-1/NUCB2 may participate in the activation of the hypothalamic-pituitary-adrenal axis in rats. Neurochem Int.

[11] Ge JF, Xu YY, Qin G, Peng YN, Zhang CF, Liu XR (2015). Depression-like behavior induced by Nesfatin-1 in rats: involvement of increased immune activation and imbalance of synaptic vesicle proteins. Front Neurosci.

[12] Geng LY, Qian FY, Qian JF, Zhang ZJ (2017). The combination of plasma glutamate and physical impairment after acute stroke as a potential indicator for the early-onset post-stroke depression. J Psychosom Res.

[13] Liu Z, Zhu Z, Zhao J, Ren W, Cai Y, Wang Q (2017). Malondialdehyde: a novel predictive biomarker for post-stroke depression. J Affect Disord.

[14] Bez Y, Ari M, Ozturk OH, Oktar S, Can Y, Sogut S (2010). Plasma Nesfatin-1 level may be associated with disease severity in patients with panic disorder. Psychopharmacology.

[15] Bez Y, Ari M, Ozturk OH, Oktar S, Can Y (2012). Increased plasma Nesfatin-1 levels in patients with obsessive compulsive disorder. Psychopharmacology.

[16] Ari M, Ozturk OH, Bez Y, Oktar S, Erduran D (2011). High plasma nesfatin-1 level in patients with major depressive disorder. Prog Neuro-Psychopharmacol Biol Psychiatry.

[17] Ding S, Qu W, Dang S, Xie X, Xu J, Wang Y (2015). Serum nesfatin-1 is reduced in type 2 diabetes mellitus patients with peripheral arterial disease. Med Sci Monit.

[18] Algul S, Ozkan Y, Ozcelik O (2016). Serum nesfatin-1 levels in patients with different glucose tolerance levels. Physiol Res.

[19] Li QC, Wang HY, Chen X, Guan HZ, Jiang ZY (2010). Fasting plasma levels of nesfatin-1 in patients with type 1 and type 2 diabetes mellitus and the nutrient-related fluctuation of nesfatin-1 level in normal humans. Regul Pept.

[20] Delitala AP, Terracciano A, Fiorillo E, Orrù V, Schlessinger D, Cucca F (2016). Depressive symptoms, thyroid hormone and autoimmunity in a population-based cohort from Sardinia. J Affect Disord.

[21] Talaei A, Rafee N, Rafei F, Chehrei A (2017). TSH cut off point based on depression in hypothyroid patients. BMC Psychiatry.

[22] Yu J, Tian AJ, Yuan X, Cheng XX (2016). Subclinical hypothyroidism after ^131^I-treatment of Graves' disease: a risk factor for depression?. PLoS One.

[23] Liu F, Yang Q, Gao N, Liu F, Chen S (2014). Decreased plasma nesfatin-1 level is related to the thyroid dysfunction in patients with type 2 diabetes mellitus. J Diabetes Res.

